# Abstract of the Procedings of the Buffalo Medical Association, Nov. 2d, 1875

**Published:** 1875-12

**Authors:** E. N. Brush

**Affiliations:** Secretary


ART. III.—*Abstract of the Proceedings of the Buffalo Medical Association, November 2d,* 1875. Reported by E. N. BRUSH, M. D., Secretary.

Dr. GOULD in the Chair.

Present—Drs. Cronyn, Gay, Little, Fowler, W. W. Miner, Rochester, Brecht, Hebenstreit, Hopkins, Folwell and Barnes.

Secretary's report read and accepted.

The application of Dr. E. C. Coxe was taken from the table and he was unanimously elected a member.

Dr. W. W. MINER read an essay on "Chronic Dislocations of the Shoulder-Joint" (see Art. II).

Dr. J. S. SMITH was appointed as Essayist for December Meeting.

Dr. GAY said that he had seen a case of dislocation of the shoulder, a few days since which occurred at Portage. The reduction had been effected by a local physician. He saw the case two weeks after; good use of the arm had been attained, but there was a depression beneath the acromion process which he attributed to stretching of capsular ligament, produced by failure to apply a bandage.

Dr. Gay also reported a case of dislocation of the left femur in a man fifty-one years old, six feet tall, and very muscular. The dislocation was into the thyroid foramen; it was reduced under ether in from twenty to thirty minutes by a process directly opposite to that employed in dislocation upon the dorsum of the illium.

Dr. CRONYN remarked that in recent dislocations into the axilla the reduction is generally easy, and is made most frequently by the heel in the axilla. With ancient dislocations the case was different The case referred to in Dr. Miner's paper as having been reduced before the curators, came under Dr. Cronyn's care in July last. He found the condition of anchylosis and deformity as described by Dr. Miner, and applied, among other means, what is known as Jarvis' adjuster, but without improvement. He obtained motion in the vicinity of the joint, but was of the opinion that in doing so he fractured the neck of the scapula; when this had united the condition was as bad as formerly. With Jarvis' adjuster you can apply any force you desire.

Manipulative measures are sometimes satisfactory and sometimes are not. Their employment is not new. The paper of Dr. Miner was full of interest, as showing how long after the accident the dislocation can be restored.

Four weeks ago he saw a gentleman, sixty years old, who fell and dislocated his shoulder; a physician was called and said he reduced the luxation; an examination revealed the head of the bone in the axilla. The luxation was readily reduced. The case illustrates the necessity of applying some retentive apparatus.

Dr. WHITE said his experience in early life was large in dislocations of shoulder-joints. Remembered one case which was in the habit, so to speak, of being dislocated. The early theory was that old dislocations could not be reduced; but he early had occasion to doubt this, and to demonstrate its falsity. Capt. Jones, sixty-three years old, came to him on the sixty-third day after dislocating his shoulder. Dr. Hamilton saw the case and confirmed the diagnosis. Succeeded in reducing the luxation by heel in the axilla. This led him to the opinon that old dislocations could be reduced. At a later period, Mrs. S., of Canisteo, came to consult him with a dislocation of ninety-six days duration. The use of heel in the axilla was ineffeetual. He then applied what is known as the Spanish windlass. This force being applied, Dr. White manipulated the arm, and in a few moments the dislocation was reduced.

He related these cases, as he had not heard the paper, in confirm ation of the grounds which he presumed Dr. Miner had taken, that chronic dislocations could be more frequently restored than was generally supposed. He had seen several which had been dislocated for over one month, but none longer than these.

Dr. ROCHESTER said that the subject reminded him of a case which he was called to treat some time since, and which was subsequently seen by Dr. J. F. Miner in consultation. A very muscular young man fell down a short flight of stairs, striking on the verge of the anus. Dr. Rochester was called on the second day, and discovering something abnormal in the position of the limb, advised that a surgeon be called, but this was objected to. After about two weeks' attendance, he insisted on calling Dr. Miner in consultation. Movement of the limb gave pain, and the thigh was held in an abnormal position. An examination was made which revealed the fact that the head of the bone was dislocated into the thyroid foramen. The introduction of the finger into the rectum confirmed this diagnosis. Under ether, reduction was made.

Dr. Folwell said he had not heard the paper, and would ask what the auther meant by chronic dislocation, whether he referred to dislocations of some time standing, or to those produced by disease.

Dr. Miner replied that he meant dislocations of long standing.

Dr. Folwell referred to Dieffenbach's case of subcutaneous section of the tendons in an old dislocation of the shoulder.

Dr. Gay read the following paper:

### SPLINTS.

On the seventh day of March, 1875, Dextrine was first introduced into the Buffalo General Hospital in the treatment of fractures. According to my best knowledge and belief, this was the first time that dextrine had been used for this purpose in this city. I had long been convinced of its utility, but from some cause or other had postponed its employment until on the day above mentioned. At a visit to the Massachusetts General Hospital during the autumn of 1867, I found dextrine there almost exclusively used for splints. Its use is certainly not new. Velpeau, I believe, was the first surgeon to recommend and employ it for splints. My first patient in the hospital upon whom dextrine was used, had fractured his left tibia one week before dextrine was used for him. His limb had been dressed with the ordinary surgical apparel. All swelling had at this time disappeared when I ordered the use of dextrine.

The article was obtained of Mr. Peabody, the druggist. Its price is so low as to enable the surgeon to use it on his poorest patients, and is so valuable as to induce him to use it also for the more fortunate and rich. Six ounces will be sufficient to make a firm splint that shall envelop the leg from the toes to the knee.

The next patient was Charles Summers, aged 26 years. He slipped on the sidewalk February 27, and fractured his left tibia and fibula at the lower third. Dextrine was not used until March

9th, when the whole circumference of the limb from the toes to the knee was enveloped in a dextrine bandage, which was so firm as to allow of the patient getting up on crutches upon the thirteenth day after injury was received.

A third patient was Lizzie Nash, aged 31 years, who on March 5th made a mis-step descending the stairway, her foot turned inward, and she fell, fracturing the left tibia at the lower third. She entered the hospital, March 9th.

Dextrine was employed on the seventh day from date of fracture. The whole limb from toes to knee was enveloped in the dextrine bandage, which enabled the patient to rise from her bed on the eighteenth day after injury, and go about with the aid of crutches.

From the time of its first employment until the present time, dextrine has been used with increasing satisfaction for most of the fracture cases that have entered the surgical wards of the hospital. When not used, sole-leather, which makes a better splint, has been used in its stead.

### PREPARATION AND MODE OF EMPLOYMENT.

The dextrine is first partially moistened with Tr. camphor; warm water is then added until the dextrine is brought to the consistence of molasses. The limb having been bandaged by a single turn of the roller, the dextrine is applied by the hand in the same way that starch is applied in the starch bandage; this done, another layer of the roller is applied, over which another coating of the dextrine is again laid, which, when dry, will be very firm. The dressing is neater, and probably more comfortable and safe; should contraction occur, if, before applying the bandages, a layer of cotton-batting be placed next the skin around the limb. If any more strength of splint is required than the dextrine alone gives, a piece of binder's board may be placed along one side of the limb between the layers of bandage, or additional layers of bandage with thicker coating of dextrine may be used.

### EXAMINATION OF THE LIMB.

If you wish to examine the seat of fracture, provided it be fracture of the leg, all you have to do is to cut the bandage or dextrine splint from the knee to the toes anteriorly. This can be done with an ordinary scalpel, but Henry's plaster-bandage cutters should be used for this purpose. There will be found sufficient elasticity in the splint to enable the surgeon to draw apart the two sides of the splint for the purpose of examination of the limb.

### SHRINKAGE OF THE LIMB.

Should the limb shrink away from the dextrine splint in which the limb is confined, a strip of the splint — say one-half inch more or less — may be cut away with the shears or cutters, and then again the borders of the two halves may be brought into apposition. Around the entire splint a roller may be placed, making the splint fit as snugly as though there had been no shrinkage of the limb. It is not necessary in making examination that the limb be moved at all.

Dr. Bartow, the House Surgeon, finding that the dextrine was liable to crumble, obviated this objection to its employment by adding a very little glue to it. The proportion of glue to dextrine being about one of the former to six ounces of the latter, and this qantity will suffice for a firm splint that shall envelop the leg from toes to the knee. Glue corrects the friability of the dextrine, while it does not lessen its resilency. A splint thus constructed may be likened to a marine shell for lightness and toughness.

### EFFECT OF HEAT.

Heat softens the splint sufficiently to enable one to bend and partially mould it. So that in order to adapt the same splint to another limb, all you have to do is to heat it, when it can readily be made to fit another limb, thus economizing material as well as time in the construction of splints.

### RELATLVE MERITS OF DEXTRINE, STARCH AND PLASTER SPLINTS

Dextrine in the manner above described, " sets " or becomes dry and firm in thirty minutes, while starch will require nearly twenty-four hours to become dry and firm. Dextrine does not "set" so soon as anhydrous gypsum, but the gypsum is very heavy and cumbersome, contrasting in this respect widely and unfavorably with the light and elegant dextrine. The expense of dextrine cannot be enough more than the expense of either gypsum or starch as to deter any one from using it, or as to induce one to prefer any other substance in its stead. Its cost at the druggist's is only thirty-five cents per pound. If glue be added, that also is cheap.

Dextrine is more convenient both for city and country practice than either gypsum or starch. It is accessible to all practitioners in city and country. Any one can prepare it for use in ten minutes, and it can be more readily washed from the hands than either gypsum or starch.

### PREPARED FOR USE.

Any one can, who likes, have a supply of splints ready for use by taking a plaster of Paris cast of the leg and moulding the splints to the cast, cutting the splint from the cast anteriorly, or both anteriorly or posteriorly, making two side splints.

### SOLE-LEATHER SPLINTS.

If there be any splint superior to the dextrine prepared in the way herein described—if I except expensive gutta percha—it is a splint made of thin sole-leather. The best splints in use since March last at the Buffalo General Hospital are those made of sole-leather. They are called the "Bartow Splint."

Dr. Bartow, the House Physician, not only introduced them, but with the assistance of a convalescent patient, manufactured them at the hospital.

They form a complete mould of the foot and leg; are either made whole, entirely enveloping the leg, or cut, so as to make side splints. In order to make these splints, it is not indispensably necessary to have metalic casts, since plaster of Paris casts of the limb will answer the purpose, provided you only wish to make a single set of splints. It is better to obtain iron castings, however, from the plaster mould. If of a leg, let the casting be of smallest sized leg. Upon this the leather, moistened, is moulded by the hammer and twisted rope. This splint, when perfectly moulded to the casting, is not removed from it, but another splint is made over this one, and the process repeated three or four times until you have splints of different sizes fitting into each other, and making a nest of splints.

The advantage of the sole-leather splint over all others is obvious. The splint is perfectly moulded to the leg, the foot to the prominences of the internal and external malleoli and corresponding depressions. The sole-leather will last any length of time, is not injured by use, and the durability of it adds to the cheapness of it. The cost of manufacturing is absolutely less than the price paid for Day's wooden splints, such as are found in market. Dr. Bartow was led to use the leather splint in the case of a one-armed man who could not use crutches when convalescing from a fractured tibia. The leg was enveloped in a well-adjusted sole-leather splint, which so firmly sustained the leg that the patient was able to rise from his bed in four weeks after injury and walk.

Sole-leather splints have been for a few years used in Bellevue Hospital, New York, but they are not perfectly moulded to the circumference of the limb, as are the splints now in use at the General Hospital.

Dr. White said that he was in the habit, forty years ago, of making splints of cloth and shellac. He was also in the habit of using leather and binder's board. He had found, however, that there was a tendency to return to wooden splints.

Dr. Cronyn said that he did not not think there was any department of surgery in which the ingenuity of the surgeon was more taxed than in the form of splints. In almost every fracture that was treated the splint had to be modified in some way to the peculiarities of the case. During the recollection of the present generation of surgeons there had been invented eight or ten different forms of splints, and yet the perfect splint had not been reached.

Dr. White said that the point had been admirably stated by Dr. Cronyn that the surgeon was better adapted fit his splint than the manufacturer.

Dr. Barnes said that he presumed nothing could be added to what had been said. The application of dextrine and leather was not new. Had had some experience in the use of these agents in the hospital. The dextrine and similar agents were liable to change in recent fractures by the change in position of the limb. The limb could not be under the supervision of the surgeon. In a recent case of ununited fracture of the tibia, the dextrine failed, but the leather was applied and held the limb firmly, and union took place.

Dr. W. W. Miner said that if the surgeon had the sense of adaptability, which was essential, he could find in almost every household materials from which to make a splint. Saw a case of a lawyer who was bound to go into court with a fracture of the tibia. It was put up in an immovable apparatus and good union resulted. Where the surgeon has the entire control of the patient he can apply these various plastic splints, and hence they find more favor in hospital than private practice.

Dr. Folwell said that he did not think that these were adaptable to compound fractures. Had one case in which it was applied to a case of suppurative disease of the knee-joint. The application being made to secure immobility of the limb, the dextrine had to be removed on account of bad odor, the pus burrowing between the limb and the splint.

Dr. Barnes related a case of compound fracture treated with dextrine, which was applied after the period of discharge had passed away. He did not wish to detract anything from the value of this form of splint, and believed that in many instances they were light, well-fitting and of sufficient strength to retain the bones well in apposition.

Dr. W. W. Miner said that a friend who had been experimenting with splints used glue and some metalic oxide, forming a splint of considerable solidity and easy of application.

Dr. Gay said that with these splints the extension could be made of the femur. Experiments had been made which showed that after the dextrine became dry it retained its shape with sufficient firmness to satisfy the demands of most cases.

PREVAILING DISEASES.

Dr. Rochester had seen much influenza of auginose variety, almost being an epidemic of croup. Had seen within the last two weeks ten or twelve cases. Had seen in consultation one case which proved fatal, the patient dying with all the symptoms of membranous croup, although he did not think that any membrane was formed.

Had found quinia very effectual, in addition to some alkali, and for this purpose employed carbonate of ammonia. Full doses of quinia were given. To a child three years old had given twelve grains in one hour; after that giving two or three grains an hour. He had been familiar with the use of quinia in croup for over twenty years, even before the publication of Dr. H. N. Eastman's article in the *N. Y. Journal of Medicine.* Had given it simply in water and sugar.

Dr. Hopkins said that Dr. Rochester's remarks had suggested to him the method in which medical men could utilize the weather reports. Before the equinoctial storm we had but little influenza, but with the appearance of the storm, the influenza appeared in horses, and later, in human families, following the track of the storm. In regard to the use of quinia in croup, he had come to the conclusion that it must be administered to get the effect, and without regard to size of dose. In one case he had administered to a child two years old sixty grains in twelve hours, during the next twenty-four, forty grains, and one hundred and twenty grains in the course of attack.

He had seen exactly the same phenomena in respiration, pulse. etc., follow the use of Verat. Verid as that observed in quinia, In both cases aqueous vapor had been used.

Dr. Rochester said that in 1854 he saw a child to whom he gave thirty grains in one night, and in twenty-four hours gave seventy-two grains. Had never used verat. verid., but had heard it well spoken of.

Dr. Gould said that he depended more upon verat. verid. than anything else; that inflammation always preceded the membranous formation, and he employed verat. verid. to arrest inflammation.

Dr. Hopkins remarked that the quinia in these large doses did not produce quininism; that in the two or three cases in which he had administered verat. verid., he had given the medicine himself, not liking to trust the attendant.

Dr. Rochester said that he could not assent to Dr. Gould's assertion that inflammation always preceded the membrane. In almost all cases of inflammatory croup an exudation was thrown out, but in the formation of true membrane, the first intimation that anything is wrong with the throat will be given by the presence of the membrane, in the majority of cases there being no inflammatory stage.

Dr. Cronyn said that he entirely agreed with Dr. Rochester. It is well known that upon a mucous surface a fibrinous deposit can not be formed. He was still of the opinion which he advanced in this Association some years since, that diptheria and true membranous croup were identical. A discussion was at the present time going on in London between Drs. Geo. Johnson, Semple, Sir Wm. Jenner, and others; one party maintaining that membranous croup was always diptheria, the other maintaining the distinct identity of the disease as membranous cronp.

Dr. Hopkins would like to ask what the nature of the process was which destroyed the mucous membrane and then gave rise to fibrinous deposit, if it was not inflammation.

Dr. Cronyn replied: a condition of irritation, fungoid or otherwise, may arise which attacking the mucous membrane of the trachea, literally dissolves or removes it without an inflammatory process, offering to the vascular suface beneath ready locality for the deposition of plastic lymph or membranous deposit.

On motion, the Society adjourned.

